# Formamide Adsorption at the Amorphous Silica Surface: A Combined Experimental and Computational Approach

**DOI:** 10.3390/life8040042

**Published:** 2018-09-23

**Authors:** Matteo Signorile, Clara Salvini, Lorenzo Zamirri, Francesca Bonino, Gianmario Martra, Mariona Sodupe, Piero Ugliengo

**Affiliations:** 1 Dipartimento di Chimica and NIS, Università di Torino, Via P. Giuria 7 — 10125 Torino and Via G. Quarello 15/A — 10135 Torino, Italy; matteo.signorile@unito.it (M.S.); clara.salvini@edu.unito.it (C.S.); lorenzo.zamirri@unito.it (L.Z.); francesca.bonino@unito.it (F.B.); gianmario.martra@unito.it (G.M.); 2 Departament de Química, Universitat Autònoma de Barcelona, 08193 Bellaterra, Catalogna, Spain; mariona.sodupe@uab.cat

**Keywords:** formamide, silica, IR spectroscopy, DFT

## Abstract

Mineral surfaces have been demonstrated to play a central role in prebiotic reactions, which are understood to be at the basis of the origin of life. Among the various molecules proposed as precursors for these reactions, one of the most interesting is formamide. Formamide has been shown to be a pluripotent molecule, generating a wide distribution of relevant prebiotic products. In particular, the outcomes of its reactivity are strongly related to the presence of mineral phases acting as catalysts toward specific reaction pathways. While the mineral–products relationship has been deeply studied for a large pool of materials, the fundamental description of formamide reactivity over mineral surfaces at a microscopic level is missing in the literature. In particular, a key step of formamide chemistry at surfaces is adsorption on available interaction sites. This report aims to investigate the adsorption of formamide over a well-defined amorphous silica, chosen as a model mineral surface. An experimental IR investigation of formamide adsorption was carried out and its outcomes were interpreted on the basis of first principles simulation of the process, adopting a realistic model of amorphous silica.

## 1. Introduction

The understanding of the origin of life from a chemical perspective is an emerging multidisciplinary topic in recent years. Originally developing in the astro- and biochemistry communities, nowadays it is spreading across other branches such as organic, physical, and computational chemistry [1]. Due to the complexity of the subject, a multifaceted approach is indeed unavoidable. Several scenarios have been hypothesized in the last decades to describe the process, from the simplest chemical moieties to biologically relevant molecules (e.g., nucleic acids, proteins, etc.), by passing through their key building blocks, such as amino acids, nucleobases, sugars, fatty acids, etc. The formation of these latter species from the simplest molecules ubiquitously found in our universe is the step where a fundamental chemical approach can contribute the most.

Among the various pathways proposed, an interesting option relies on the polyhydric chemistry of formamide: as reported in several research papers [2,3,4,5,6,7,8,9,10,11,12], formamide is able to originate many relevant prebiotic molecules (particularly nucleobases) under mild thermal or photochemical reaction conditions. Furthermore, the selectivity toward specific products is strongly affected by the presence of mineral phases in the reaction environment; therefore, it is logical to expect that the surfaces of these minerals could drive the reaction steps from bare formamide to final products, eventually also catalyzing some of these processes. Surprisingly, such effect on selectivity is observed also for extremely simple earth-abundant minerals, e.g., carbonates, aluminates, silicates, etc. [10]. In the latter category, bare silica has also been shown to drive the reactivity of formamide toward specific products (mostly purine and cytosine), whereas it has been found to be inert toward many industrially exploited chemical processes (where it is effectively exploited as a catalyst support).

Even if the reactivity of formamide at the silica surface has been demonstrated and documented [13], a physicochemical description of the fundamental steps of this process is missing in the literature. This is not limited to the study of reactivity, but also includes the study of the bare formamide–SiO_2_ interaction (i.e., adsorption). Even if it can be considered straightforward, the study of formamide adsorption on silica presents some criticisms that have to be addressed in order to provide a rigorous and clean description of the process. First of all, silica is a rather general label describing thousands of different polymorphs, both crystalline and amorphous. Then, depending on the “chemical history” of each sample, surface properties (exposed faces, density of terminal hydroxyl groups, etc.) can vary greatly on formally identical materials. A further complication arises with formamide, which is known to favorably form dimers, thus increasing the variability on the species reaching the surface in a typical gas-phase adsorption experiment. The combination of all these variables can potentially lead to significant heterogeneity in experimental results, thus a precise experimental design is required in order to get proper insight into the formamide adsorption process on silica. By reducing the variability of the system under study, its theoretical modelling is facilitated too, since well-defined systems are obviously easier to simulate with fully representative models.

In this work, we adopted the well-known Aerosil OX 50 (A50) pyrolitic silica as a model. Despite its amorphous structure, A50 has a significant surface area (50 m^2^ g^−1^, according to the producer) compared to commercially available crystalline SiO_2_, with a relatively low surface concentration of hydroxyl groups, i.e., silanols. Through ad hoc thermal treatments [14], the population of the latter can be finely tuned toward a large prevalence of isolated silanols, thus giving rise to a well-defined surface. Accounting for these peculiarities, A50 represents an optimal model of silica in the framework of a fundamental adsorption study. Nevertheless, the IR spectra of formamide adsorbed on A50 revealed heterogeneous behavior. However, on the basis of only one experiment, it is impossible to ascribe these small spectral differences to the adsorption of formamide on slightly different sites and/or to different ways of interaction of the molecule at the same place on the surface. In order to fully elucidate the adsorption mechanism, a computational study was performed, aiming to predict the interaction at different sites with various molecule–surface reciprocal orientations. Furthermore, the effect of formamide loading was considered by simulating single and double adducts. IR spectra were simulated for each structure within the harmonic approximation and combined through a Boltzmann’s weighted average to give a reconstruction of the experimental one.

## 2. Materials and Methods

### 2.1. Experimental Approach

Aerosil 50 (A50) was chosen as the model amorphous silica, since it is a well-known material whose physicochemical properties and interactions with several biologically relevant molecules have been studied in depth [15,16,17]. Pristine A50 was preliminary treated in order to obtain a controlled and homogeneous surface silanol distribution. The method proposed by Rimola et al. was applied [14], thus exposing A50 to 2-step thermal activation in a static furnace. During the first step, the bare silica (in self-supported pellet form) was heated to 450 °C at a rate of ~10 °C min^−1^, with the temperature maintained for 2.5 h. These procedures yield a silica surface with a final silanol density of ~1.5 isolated OH per nm^2^ and with a negligible amount of interacting OH group. The already pelletized sample was directly used in the *in situ* IR study of formamide adsorption. We adopted a home-made quartz glass spectroscopy cell with minimal optical path and equipped with KBr windows. After 0.5 h of outgassing at room temperature (RT), the sample was exposed for 1 h to the vapor pressure (~1.5 mbar) of formamide at the same temperature. After this loading stage, the desorption of formamide from the silica surface upon outgassing at RT was monitored. IR spectra were collected with a resolution of 2 cm^−1^ on a Bruker Vertex 70 Fourier-transform spectrometer equipped with a cryogenic mercury–cadmium–telluride (MCT) detector operated at liquid N_2_ temperature (77 K).

### 2.2. Computational Approach

**Figure 1:**
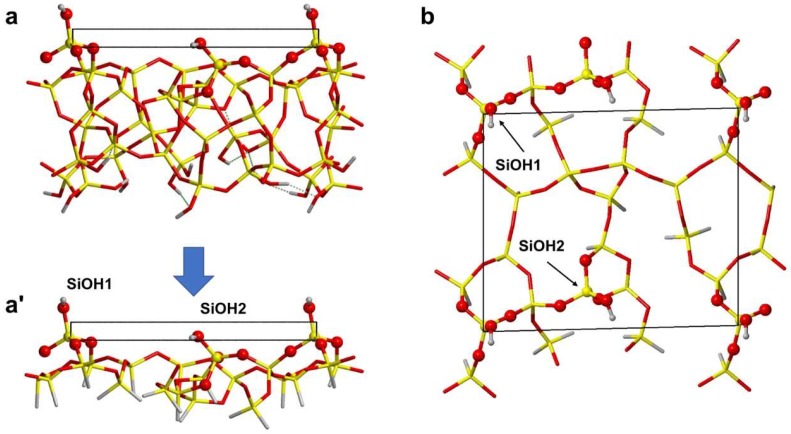
Side views of: (**a**) reference SiO_2_ model from [23] and (**a’**) the derived thin SiO_2_ model adopted in this work; (**b**) top view of the thin SiO_2_ model.

In order to simulate the adsorption of formamide over A50, a periodic 2-dimensional slab silica surface model with hydroxylation similar to the experimental one (~1.5 OH/nm^2^) was derived. With respect to previously proposed models [13,18,19,20,21,22,23], a thinner one was adopted in this work. In detail, the thickness of the model previously proposed by Ugliengo et al. [23] was stepwise reduced down to a single monolayer of SiO_2_. On the cut side, the surface was terminated by silane groups, keeping the position of terminal H fixed at the original coordinates of the former O atoms. The procedure is schematically highlighted in Figure 1.

**Figure 2:**
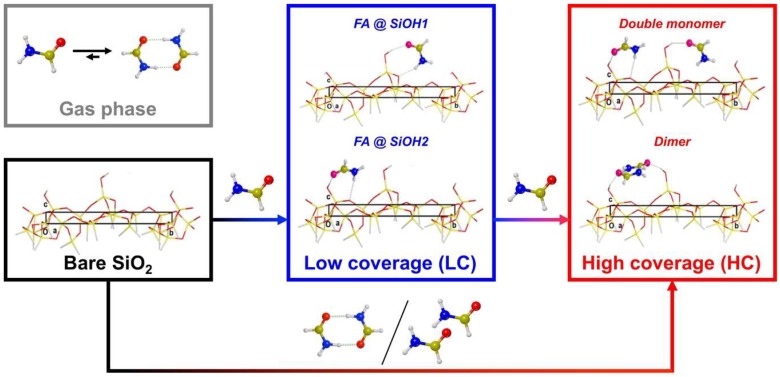
Scheme accounting for all cases considered of formamide adsorbed at the silica surface.

While the OH termination seems more physically meaningful, it has been shown in the past (see, for instance, [13]) that processes occurring at silanol groups far away from the terminal frontier (in this case, the back region of the slab) are essentially insensitive to the kind of termination (Si–H or Si–OH). Furthermore, compared to the OH termination, imposing the constraints on the H terminal atoms to restore the structural memory of the missing silica bulk is cleaner and simpler. The resulting surface exhibits 58 atoms (including the 14 terminal H) with unit cell parameters *a* = 11.64 Å, *b* = 13.60 Å, and ***γ*** = 88.65° fixed at those optimized for the larger silica model, while the atomic coordinates of all atoms (except the terminal H atoms of the silane groups) were fully relaxed. The model sports 2 different isolated silanols, hereafter referred to as SiOH1 and SiOH2 (see Figure 1). The main difference between these is greater protrusion of SiOH1, whereas SiOH2 lies closer to the surface. We docked formamide to the silica surface in order to simulate the most relevant adsorption processes by maximizing H-bond interactions and fully optimized the coordinates of all atoms, keeping the unit cell size fixed at that of the free silica slab (as well as the H atoms of the silane groups). Since these H-bond interactions usually fall in the energy range proper for physisorption, dispersive interactions compete to determine both the final structure and the extent of the interaction energy of each adduct and, ultimately, the vibrational spectra [24]. Therefore, the final optimized structures result from a delicate balance between these 2 components of the interaction energy. Furthermore, since 2 different adsorption sites (i.e., silanols) are exposed in the unit cell, coverage must also be considered: for simplicity, we refer to low coverage (LC) for a single formamide adsorbed on the surface and high coverage (HC) when 2 molecules are adsorbed (i.e., formamide interacts with all silanols). Another relevant aspect is that formamide dimerizes with high interaction energy [25,26], thus the adsorption of dimeric species was also considered. At our computational level (*vide infra*), the Gibbs free energy for the dimerization reaction in the gas phase is −18 kJ mol^−1^, illustrating the prevalence of the dimeric over isolated formamide in the gas phase. While the work by Mardyukov et al. [25] on formamide dimers reports at least 5 different possible conformers, here we focused exclusively on the most stable adduct (case A, Table 1 in [25]), as its population in the gas phase will dominate the distribution of conformers. Furthermore, at least 2 formamide molecules can adsorb simultaneously at the surface, directly yielding an HC structure. Moreover, at the HC condition, each formamide molecule can interact with a silanol (in a “double-monomer” fashion) or can give rise to adsorbed dimeric structures, with a strong molecule–molecule interaction in the latter case. Figure 2 shows the general scheme accounting for all possible ways in which formamide can dock to the silica surface.

Due to the complexity of the adsorption process, the study reported in this work is limited to the simplest case of subsequent adsorption of single formamide (FA) molecules, according to the following processes:

SiO_2_ + FA → SiO_2_⋯FA (1)

SiO_2_⋯FA + FA → SiO_2_⋯2(FA) (2)

Interaction energy (Δ*E*), enthalpy (Δ*H*), and Gibbs (Δ*G*) free energy for all considered processes were computed with the usual formula:

(3) $$\Delta X=X_{products}-X_{reactants}$$

where *X = E, H,* or *G.* Products refer to the right-hand side of Equations (1) and (2) (adsorbed complexes), therefore the computed ΔX are all negative for bounded adducts at silica surfaces.

According to this stepwise process, 12 adsorption processes were simulated, 5 LC and 7 HC (of which 4 were dimeric and 3 were double-monomeric structures). A detailed structural overview of these models is given in the Supplementary Materials (Figures and Tables S1 to S12).

All the calculations presented in this work were performed with CRYSTAL17 [27,28], adopting the Perdew–Burke–Enzerhof (PBE) generalized gradient approximations (GGA) functional for both correlation and exchange terms [29]. As the basis set, Si and O atoms are described through the polarized double-ζ quality basis set proposed by Nada et al. [30], whereas formamide (H, C, N, O) and terminal silane groups use the Ahlrichs valence triple zeta (VTZ) basis set (including polarization functions) [31]. The choice of a richer basis set for formamide (Ahlrichs VTZ) with respect to the one adopted in the description of the SiO_2_ surface is intended to limit the computational cost of the calculation while keeping the best electrostatic and polarizability description of formamide within the computational limits. Improving the formamide basis set is also beneficial in reducing the basis set superposition error (BSSE), shown to be rather small for the Ahlrichs family of basis sets. This is particularly important when considering that H-bond interactions compete with dispersion interactions of formamide with silica surface, which requires a well-balanced description of both surface and molecule contacts. The electron density was integrated over a pruned grid with 75 radial points and a maximum 974 angular (defined as XLGRID within the code). The truncation criteria for bielectronic integrals, i.e., the TOLINTEG parameters, were set to {7 7 7 7 16}, pursuing good accuracy. Diagonalization of the Hamiltonian matrix was performed at Γ point only (SHRINK parameter set to {1 1}). The Grimme D2 empirical scheme was applied to consider the effect of dispersive interactions [32]. As this term is simply added to the electronic energy, the purely dispersive contribution to the interaction energy Δ*E* (Equation (3)) was easily extracted. All the computational parameters not mentioned above were left as default values according to the CRYSTAL17 manual [33]. Adsorption energies were calculated after energy relaxation upon formamide adsorption and corrected for the BSSE according to the counterpoise method [34]. Adsorption enthalpy, Gibbs free energy, and vibrational frequency for formamide (including IR intensity) were estimated through a reduced Hessian approach, where the calculation was limited to a fragment envisaging the adsorbed molecule(s) and the silanol groups (all of them were always included in the fragment for which frequencies were computed, irrespective of their occupation by formamide). Vibrational frequencies for the C=O stretching and NH_2_ bending (as the main target of the experiments) were scaled by 2 separate proportionality factors, 0.9972 and 1.0102, respectively, derived from the ratio of experimental to calculated frequencies of the modes for the free formamide molecule [35]. This accounts for anharmonicity and facilitates a direct comparison with the experimental adsorption bands.

## 3. Results

### 3.1. FTIR of Formamide Adsorbed on A50

**Figure 3:**
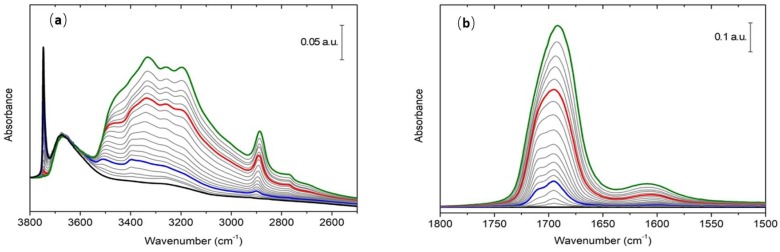
(**a**) IR spectra of formamide adsorbed on the A50 surface in the X–H (X = C, N, or O) stretching region; and (**b**) baseline subtracted IR spectra of formamide adsorbed on the A50 surface in the carbonyl stretching region. Black curves refer to bare silica outgassed 0.5 h at room temperature (RT). Green curves refer to maximum formamide coverage achieved after 1 h of exposure of silica to its vapor pressure (1.5 mbar) at RT. Red represents the high coverage (HC) reference spectrum. Blue curve represents the low coverage (LC) reference spectrum. Gray curves were obtained during the outgassing of formamide at RT, and their time evolution is from green curves toward black ones.

Figure 3 shows the FTIR spectra of formamide adsorbed on the silica surface in the most interesting spectral regions, i.e., where the carbonyl stretching and X–H (X = C, N, or O) stretching modes fall.

In the X–H (X = C, N, or O) stretching modes region, if we look at the spectrum before formamide contact (black curve of Figure 3a), the dominant feature is a sharp peak with maximum at 3748 cm^–1^. This signal is straightforwardly ascribable to isolated silanols exposed at the silica surface. A second feature is represented by a broader band peaking at ~3675 cm^–1^, showing the presence of a fraction of silanols interacting through hydrogen bond. At maximum formamide dosage, the former signal is fully eroded, suggesting that the molecule has direct interaction with isolated silanols, as logically expected. Conversely, the latter band seems unperturbed upon adsorption, pointing out that related interacting silanols are not accessible by formamide: these are most probably intraglobular silanol nests, thus chains of hydrogen bonded silanols embedded in inaccessible internal cavities of the silica particles. Another relevant feature observed at full coverage is a complex overlap of signals in the 3000–3500 cm^−1^ range due to the formamide vibrational modes, also establishing hydrogen bonds with the SiO_2_ surface and/or other formamide molecules.

Due to the effect of the hydrogen bond, the X–H band is very broad and poorly informative of the specific interaction with formamide. Its broadness indicates that H-bond is occurring between formamide and silanol groups at the surface, as expected. Different spectral windows are therefore more effective in characterizing the interacting formamide. In this regard, the observation of the carbonyl stretching region is much more informative. This latter range at maximum coverage (red curve of Figure 3b) is characterized by the presence of a complex band centered at approximately 1690 cm^−1^, most probably arising from the overlap of more than a single component, as suggested by its broad, asymmetric shape. Looking at the outgassing sequence, two distinct components can be recognized: the most intense blue one shifts from 1690 cm^−1^ up to 1695 cm^−1^ while coverage is reduced, whereas the second component appears as a shoulder at 1710 cm^−1^, becoming more defined at lower coverages and with a frequency that remains constant at every coverage. Both of these signals can be related to carbonyl stretching modes, and since they are considerably red-shifted with respect to the carbonyl stretching frequency of the gas-phase formamide (1754 cm^−1^ [35]), they most probably relate to the molecule interacting with different sites or generating different adduct structures at the silica surface. In general, probing acid surface sites with molecules containing the weakly basic carbonyl group is a well-established tool in the field of vibrational spectroscopy [36,37,38]. The interaction of the carbonyl group with the surface sites leads to a perturbation of its electronic structure, which produces a shift of its stretching mode strongly correlated to the strength of the interaction. Thus, the local structure of the surface sites and strength of their interaction with the probe molecule can be inferred [38]. In the same spectroscopic region, a weak and broad band ascribed to the NH_2_ bending mode is observed (Figure 3b). At maximum coverage, it is centered at ~1605 cm^−1^ and progressively red-shifts down to 1600 cm^−1^ upon outgassing. No clear components are revealed during formamide desorption as occur for the carbonyl stretching band.

### 3.2. Simulation of Formamide-A50 Adducts

#### 3.2.1. Low Coverage Models

Table 1 shows the energetic values for the adsorption of formamide at low coverage on the SiO_2_ surface.

**Figure 4:**
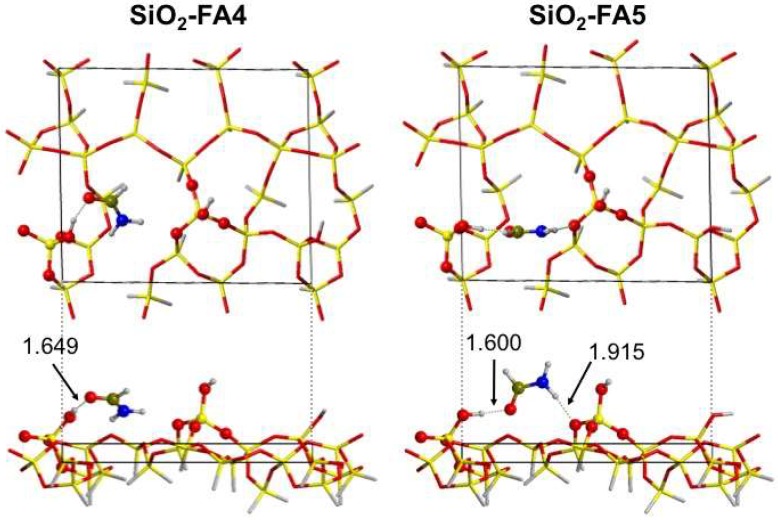
Top and side views of the most stable low-coverage models, SiO_2_–FA4 and SiO_2_–FA5. Also shown are the relevant formamide–surface distances (in Å).

Independently from the model (i.e., the type of formamide adduct at the SiO_2_ surface), the adsorption process is exothermic, according to the negative values of ΔE^c^ and ΔH^c^. Nevertheless, they assume rather different values, in agreement with the variety of interactions formamide can establish with the surface. In all cases (except for the SiO_2_-FA2 model), the key interaction is represented by the H-bond from a silanol group at the surface and the carbonyl group of formamide. Additionally, the same structures further present a second H-bond interaction from the amine group of formamide toward siloxane bridges at the surface. For all considered structures, the NH_2_ group always behaves as a weak Brønsted acid and is never involved as an H-acceptor group. Despite the analogous docking, the ΔE^c^ values are rather different among these four structures; an important parameter to be considered is the extent of the dispersive forces, as highlighted in Table 1. In fact, some of the adducts are strongly stabilized by this energetic term, e.g., SiO_2_-FA3 and SiO_2_-FA4. Looking at the spatial relation between formamide and SiO_2_ surface, in these models the molecules lean flat at the surface, maximizing their dispersive interaction. Conversely, in models SiO_2_-FA1 and SiO_2_-FA5, dispersive interaction is lower according to the “perpendicular” arrangement of formamide with respect to SiO_2_. As an example, a graphic comparison of adducts SiO_2_-FA4 and SiO_2_-FA5 is shown in Figure 4.

Finally, SiO_2_-FA2 represents an extreme case, where the formamide–surface interaction can be considered barely dispersive, as the electronic component of ΔE^c^ accounts for less than 40% of the total. Interestingly, formamide establishes the stronger adduct (SiO_2_-FA4) with the less exposed silanol group, most probably because of the easier formation of a weak H-bond between the amine group and a distorted Si–O–Si bridge (see Figure 4) and the efficient dispersive interaction due to the molecule sitting closer to the surface. The computed Gibbs free energy of interactions shows the formation of adducts involving direct H-bonds as spontaneous. The lowest ΔG^c^ (stronger affinity) are achieved for the models where formamide interacts with the less exposed silanol (e.g., SiO_2_-FA4 and SiO_2_-FA5). Translating the Gibbs free energy to the probability of formation of each adduct on the basis of the Boltzmann distribution function, the latter accounts for the 97% of low-coverage adsorption structures. Finally, the SiO_2_-FA2 adduct in which no specific H-bond is present (see Supplementary Figure S2) is the only case exhibiting a positive ΔG^c^, verifying the key role of H-bond in making the adsorption process spontaneous.

#### 3.2.2. High-Coverage Models

A set of high-coverage models was generated by docking a second formamide molecule toward the previously considered structures in which one formamide was already preadsorbed. Their interaction energy with respect to the most stable adduct at low coverage (i.e., SiO_2_-FA4) was calculated. With respect to the low-coverage situation, higher variability is possible for the high-coverage case. The second molecule can interact with the silanol not yet bound upon the first adsorption process, but, at the same time, it can also establish a direct lateral interaction with the previously adsorbed formamide. In this case, a dimer-like adsorbed species will be formed. Conversely, if the second molecule only interacts with the surface without direct involvement of the previously adsorbed one, a double-monomer adduct can be identified. Accounting for this further degree of freedom and for further orientations, a set of seven high-coverage structures was built (of which three were double monomers and four were dimers). The energetic parameters for adsorption of the second formamide molecule are reported in Table 2. For the sake of comparison, the same energetic values, but computed for the direct adsorption of two formamide molecules over the pristine SiO_2_ surface, are given in Supplementary Table S13.

**Figure 5:**
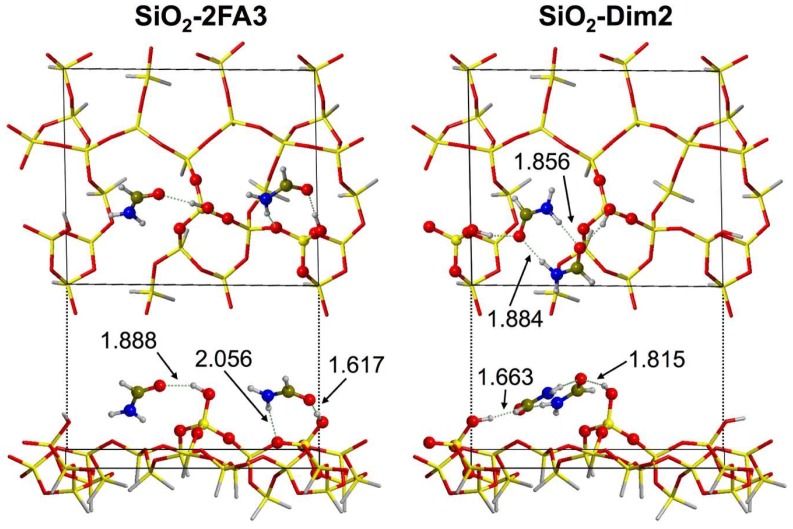
Top and side views of the most stable high-coverage models, SiO_2_-2FA3 and SiO_2_-Dim2. Also shown are the relevant formamide–surface distances (in Å).

The adsorption of a second formamide molecule is exothermic for all considered high-coverage structures, with average values of ΔE^c^ and ΔH^c^ just slightly lower than those for single adsorption (see Table 1). In detail, most of the adducts exhibited ΔE^c^ in the −45/−60 kJ mol^–1^ range, whereas in only two cases stronger interactions occurred, the double-monomeric SiO_2_-2FA3 and the dimer-like SiO_2_-Dim2 models. The structure of the latter is shown graphically in Figure 5.

In the case of the SiO_2_-2FA3 double-monomer adduct, each formamide molecule binds to a silanol group, forming a chain-like structure along the surface. The comparison with SiO_2_-FA4 shows that the adsorption does not follow the Langmuir model, as the ΔG^c^ values are 21 and 18 kJ mol^−1^, respectively. This indicates some sort of cooperation between adsorption sites (as shown in Figure 5 for SiO_2_-2FA3) due to bridging interactions caused by the adsorbed formamide. Instead, in the SiO_2_-Dim2 dimeric adduct, the two formamide molecules interact with a reciprocal orientation resembling the free dimer in the gas phase. Moreover, further H-bonds are established between carbonyl groups and silanols, securing the dimeric structure to the surface. As previously commented for the SiO_2_-FA4 low-coverage adduct, in both structures described here, the formamide molecules lean almost flat at the surface, maximizing the dispersive interactions. Even if the SiO_2_-2FA3 structure does not present the highest stability (SiO_2_-Dim2 has higher ΔE^c^ and ΔH^c^ in absolute value), its ΔG^c^ value is the lowest, probably due to a larger entropic cost for SiO2-Dim2 due to the formation of the constrained dimeric structure. Therefore, the SiO_2_-2FA3 Boltzmann population strongly dominates (91%) the HC case. It is worth noting that the same Boltzmann population results when considering the ΔG^c^ values computed, referring to the direct adsorption of two formamide molecules at the bare SiO_2_ surface to yield the HC adducts (see data in Supplementary Table S13).

### 3.3. Simulation of FTIR Spectra of Formamide-A50 Adducts: Comparison with Experimental Results

**Figure 6:**
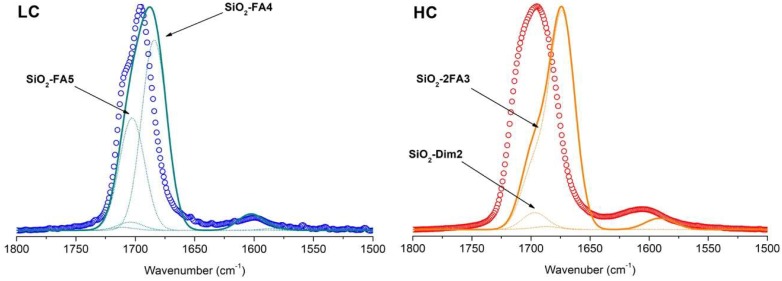
Reference experimental spectra at low coverage (LC, empty blue dots) and high coverage (HC, empty red dots) compared to the simulated ones (solid cyan and orange lines for LC and HC respectively). Simulated spectra were reconstructed from the computed vibrational frequencies (spectral profiles described by Gaussian functions with full width at half maximum (FWHM) of 20 cm^−1^) for each LC/HC adduct weighted over the corresponding Boltzmann population. The single simulated spectral components (i.e., each belonging to a single adduct model) are reported as dotted lines for both LC and HC (nil components are omitted for clarity).

By means of the results for the model described in the previous paragraphs, a tentative interpretation of the experimental IR spectra was carried out. The simulated spectrum for each adduct was built by overlapping Gaussian functions whose main parameters (maximum position and intensity) were estimated through a reduced Hessian frequency calculation. The frequencies were scaled as described in Section 2.2. The full width at half maximum (FWHM) of Gaussian functions was arbitrary set to 20 cm^−1^. The overall simulated spectra were computed by a linear combination of the spectra of each adduct (including only the single adducts for the LC case and, conversely, only double adducts in the HC case), weighted by the corresponding Boltzmann populations (see Table 1 for LC and Table 2 for HC). The intensity of the overall simulated spectra was normalized to the maximum absorbance of the corresponding experimental reference spectra to facilitate the comparison. Results of this procedure are graphically outlined in Figure 6.

For the LC case, a nice qualitative agreement is found between the reference experimental spectrum and the simulated one. The clear bimodal shape of the absorption line in the experimental spectrum is effectively reproduced by modelling, and the two observed spectral components can be assigned to specific formamide adducts at the silica surface, as revealed by quantum mechanical simulation. In particular, the main feature peak at 1695 cm^−1^ is assigned to the SiO_2_-FA4 model (see Figure 4) with formamide interacting with the silanol group lying closer to the silica surface. The formamide molecule is further oriented with its symmetry plane parallel to the surface, thus maximizing the dispersive interaction. The high-frequency shoulder of the peak (roughly centered at 1710 cm^−1^) is instead ascribed to the SiO_2_-FA5 adduct (see Figure 4), where formamide interacts with the same silanol taking part as the FA4 adduct, but with its symmetry plane perpendicular to the surface. The NH_2_ bending mode feature at roughly 1600 cm^–1^ is well reproduced by simulation too, further validating the reliability of the combined experimental–simulation approach for the LC case.

In the HC case, a less satisfactory description of experimental outcomes is achieved by using spectral information from the simulation. The overall simulated spectrum (which is dominated by the contribution of SiO_2_-2FA3 adduct) is red-shifted too much with respect to the experimental one, while components giving closer absorption frequencies (such as SiO_2_-Dim2, the second adduct in terms of stability) are suppressed by the low value of their Boltzmann population. In detail, the maximum adsorption for the carbonyl stretching mode in the simulated spectrum is centered at 1675 cm^–1^, falling ~20 cm^−1^ below the experimental maximum. A similar red-shift also resulted in the NH_2_ bending. However, the line shape of the simulated carbonyl peak closely resembles the experimental one, with a clear shoulder falling at higher frequencies with respect to the peak maximum. Since the main difference between simulated and experimental spectra is represented by a frequency offset, it may be possible that such discrepancy derives from incorrect scaling of the calculated frequency when formamide experiences multiple interactions (as in the HC case). Still, the possibility that more complex adsorption structures (e.g., involving “liquid like” and/or multiple layers of formamide) are involved at HC cannot be *a priori* excluded.

## 4. Conclusions

The adsorption of formamide on silica characterized by low hydroxylation (about 1.5 silanol groups/nm^2^) was investigated through a combined experimental–simulation approach, in which experimental IR spectroscopic features were interpreted by means of a quantum mechanical modeling study carried out on a realistic amorphous silica slab model. The IR spectra showed a lowering of the C=O stretching frequency, a clear indication of an H-bond interaction between isolated silanol groups and adsorbed formamide. Nonetheless, the C=O stretching band has a squat-like shape, clearly indicating a rather complex scheme of interaction with formamide molecules, not easily interpreted on a single kind of adsorption site, despite the nature of isolated silanol groups at the considered surface. A possible explanation of the observed spectral features is provided by the computer modeling results, in which the adopted surface silica model exhibits two isolated silanol groups with different spatial accessibility and surroundings. Adsorption of formamide was simulated by docking formamide toward the silica surface model in a stepwise approach, from low coverage (LC, 1 formamide per unit cell) to high coverage (HC, 2 formamides per unit cell). A rather ample number of interaction cases were studied, all of them fully characterized as far as optimum structure, Gibbs free energy of adsorption, and vibrational spectra in the harmonic approximation. Analysis of the results reveals that H-bond interaction is the leading component of the interaction energy. However, dispersion interaction is always a significant component of the final interaction energy and cannot be ignored in calculations of this kind. Interestingly, cases in which H-bond is absent and the interaction is exclusively dominated by dispersion component exhibit positive Gibbs free energy of adsorption (unbound case). The infrared spectrum in the C=O and NH_2_ region was simulated using the computed harmonic frequencies and intensities through a Boltzmann weighted sum of associated Gaussian-type functions to simulate the natural bandwidth. For the LC case, remarkably good agreement with the experimental spectrum was achieved. Far less satisfactory was the reconstruction of the HC spectrum. The reason for that may be the insufficient coverage attained by the simple HC cases or even the simple scaling of the C=O and NH_2_ frequencies not accounting for H-bond.

The present results allow extension of this study to the role of silica in the chemical reactivity of formamide, i.e., its decomposition in HCN and H_2_O, a crucial step for further chemical evolution toward nucleic base synthesis. Studies both experimental and computational along that direction are already in place in our laboratory.

## Figures and Tables

**Table 1:** Adsorption energy (ΔE^c^, with explicit dispersive contributions in parentheses), enthalpy (ΔH^c^), and Gibbs free energy (ΔG^c^) for the SiO_2_–formamide adduct low-coverage models. Energy values are calculated according to Equation (1) (*vide supra*). Interaction energy values (in kJ mol^–1^) have been basis set superposition error (BSSE) corrected by the counterpoise method. The last column shows the Boltzmann population (*p* ≤ 1) calculated from ΔG^c^ at standard temperature and pressure (STP) conditions (298.15 K, 1 atm).

Model	ΔE^c^ (ΔE disp)	ΔH^c^	ΔG^c^	*p*
SiO_2_-FA1	−69.9 (−18.8)	−62.8	−13.3	0.03
SiO_2_-FA2	−44.0 (−28.1)	−38.1	8.6	0.00
SiO_2_-FA3	−68.0 (−26.4)	−60.7	−9.6	0.01
SiO_2_-FA4	−80.5 (−27.4)	−74.2	−21.3	0.67
SiO_2_-FA5	−63.3 (−15.6)	−56.8	−19.3	0.30

**Table 2:** Adsorption energy (ΔE^c^, with explicit dispersive contributions in parentheses), enthalpy (ΔH^c^), and Gibbs free energy (ΔG^c^) for the SiO_2_–formamide adduct high-coverage models. Energy values are calculated according to Equation (2) (*vide supra*). Energy values (in kJ mol^–1^) have been BSSE corrected through the counterpoise method. The last column shows the Boltzmann population (*p* ≤ 1) calculated from ΔG^c^ at STP conditions (298.15 K, 1 atm).

Model	ΔE^c^ (ΔE disp)	ΔH^c^	ΔG^c^	*p*
SiO_2_-2FA1	−57.6 (−28.4)	−50.1	−1.7	0.00
SiO_2_-2FA2	−56.4 (−20.4)	−49.2	−6.3	0.01
SiO_2_-2FA3	−67.3 (−16.9)	−59.9	−17.9	0.91
SiO_2_-Dim1	−46.8 (−24.2)	−39.9	7.2	0.00
SiO_2_-Dim2	−73.5 (−24.1)	−64.7	−12.0	0.08
SiO_2_-Dim3	−51.7 (−19.3)	−43.4	2.3	0.00
SiO_2_-Dim4	−58.5 (−21.3)	−49.4	−1.5	0.00

## Supplementary Materials

The following are available online at http://www.mdpi.com/2075-1729/8/4/42/s1, Figures S1 to S12: graphical details of the 12 formamide adsorption models, Tables S1 to S12: key structural and spectroscopic features for the 12 formamide adsorption models, Table S13: Adsorption energies, enthalpies and Gibbs free energies for the SiO_2_-formamide adducts high coverage models generated by double adsorption over the pristine SiO_2_ surface.
