# Supplementary material for: Formamide Adsorption at the Amorphous Silica Surface: A Combined Experimental and Computational Approach
Source: Life (Basel). 2018 Sep 23;8(4):42. doi: 10.3390/life8040042 (PMC6316577; doi:10.3390/life8040042)
Supplement: Supplementary file 1 [file life-08-00042-s001.pdf]

Supplementary Material for

Formamide adsorption at the amorphous silica surface: a combined experimental and computational approach

Matteo Signorile [1], Clara Salvini [1], Lorenzo Zamirri [1], Francesca Bonino [1], Gianmario Martra [1], Mariona Sodupe [2], Piero Ugliengo [1*]

[1] Dipartimento di Chimica and NIS, Università di Torino, Via P. Giuria 7 – 10125 Torino and Via G. Quarello 15/A – 10135 Torino, Italy

[2] Departament de Química, Universitat Autònoma de Barcelona, 08193 Bellaterra, Spain

* Correspondence: piero.ugliengo@unito.it; Tel.: +39-011-670-4596

Detailed structural information for the adducts commented in the main text are provided hereafter (Figures and Tables S1 to S12)

SiO_2–FA1

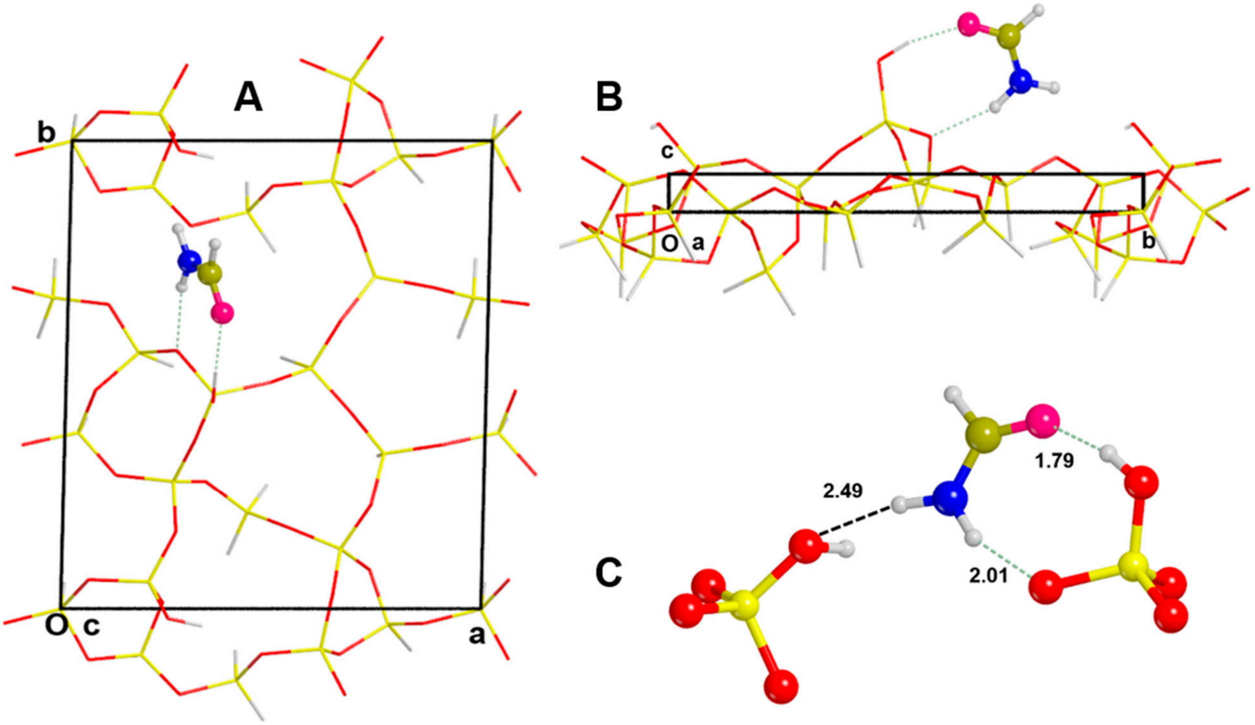

Figure S1. Relaxed geometry of the SiO_2–FA1 model: **(A)** top view; **(B)** side view (along *a* axis); and **(C)** detail of specific interaction and key distances (given in Å). Formamide oxygen is reported in pink, carbon in dark yellow, nitrogen in blue, hydrogen in gray, silicon in bright yellow, SiO_2 oxygen in red.

Table S1. Key structural and spectroscopic features for the SiO_2–FA1 model. Distances are given in Å, frequencies in cm^{-1}. The ν(C=O) and δ(NH_2) frequencies have been scaled with separated scale factors.

**SiO_2–FA1**	
d(C=O)	1.235
ν(C=O)	1704
δ(NH_2)	1582

SiO_2–FA2

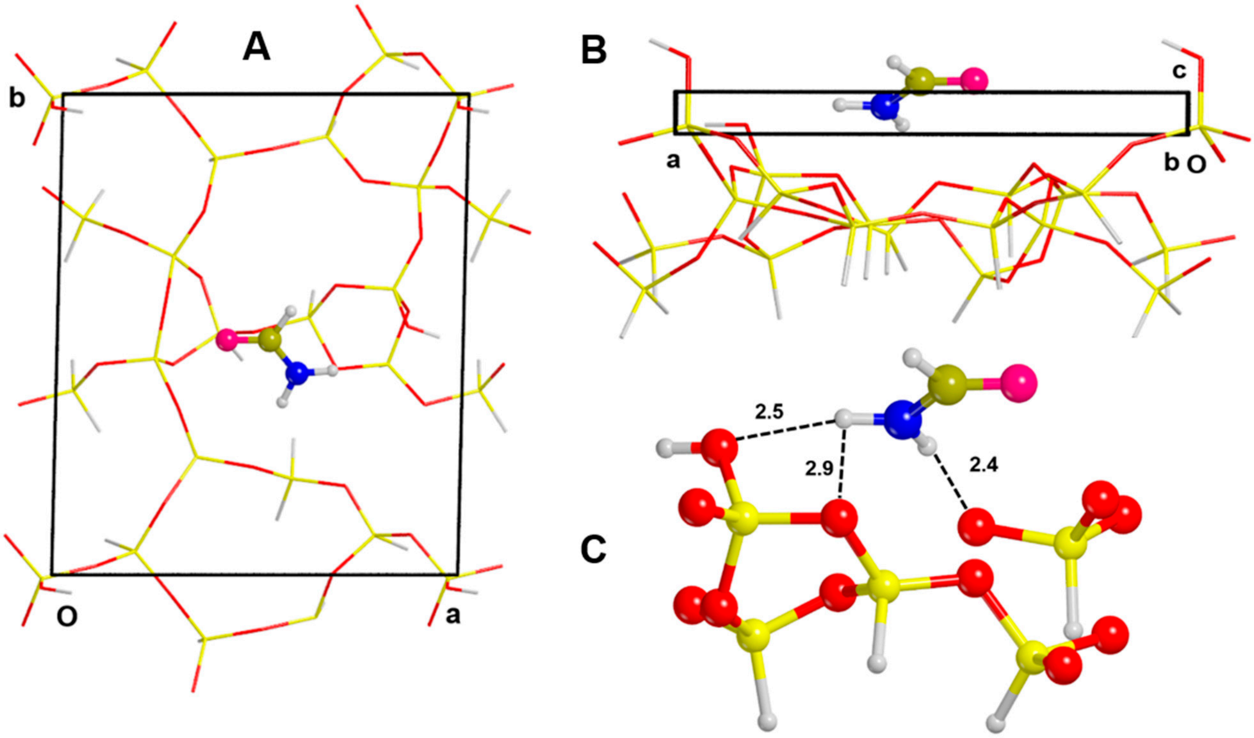

Figure S2. Relaxed geometry of the SiO_2–FA2 model: **(A)** top view; **(B)** side view (along *a* axis); and **(C)** detail of specific interaction and key distances (given in Å). Formamide oxygen is reported in pink, carbon in dark yellow, nitrogen in blue, hydrogen in gray, silicon in bright yellow, SiO_2 oxygen in red.

Table S2. Key structural and spectroscopic features for the SiO_2–FA2 model. Distances are given in Å, frequencies in cm^{-1}. The ν(C=O) and δ(NH_2) frequencies have been scaled with separated scale factors.

SiO_2–FA2	
d(C=O)	1.226
ν(C=O)	1724
δ(NH_2)	1591

SiO_2–FA3

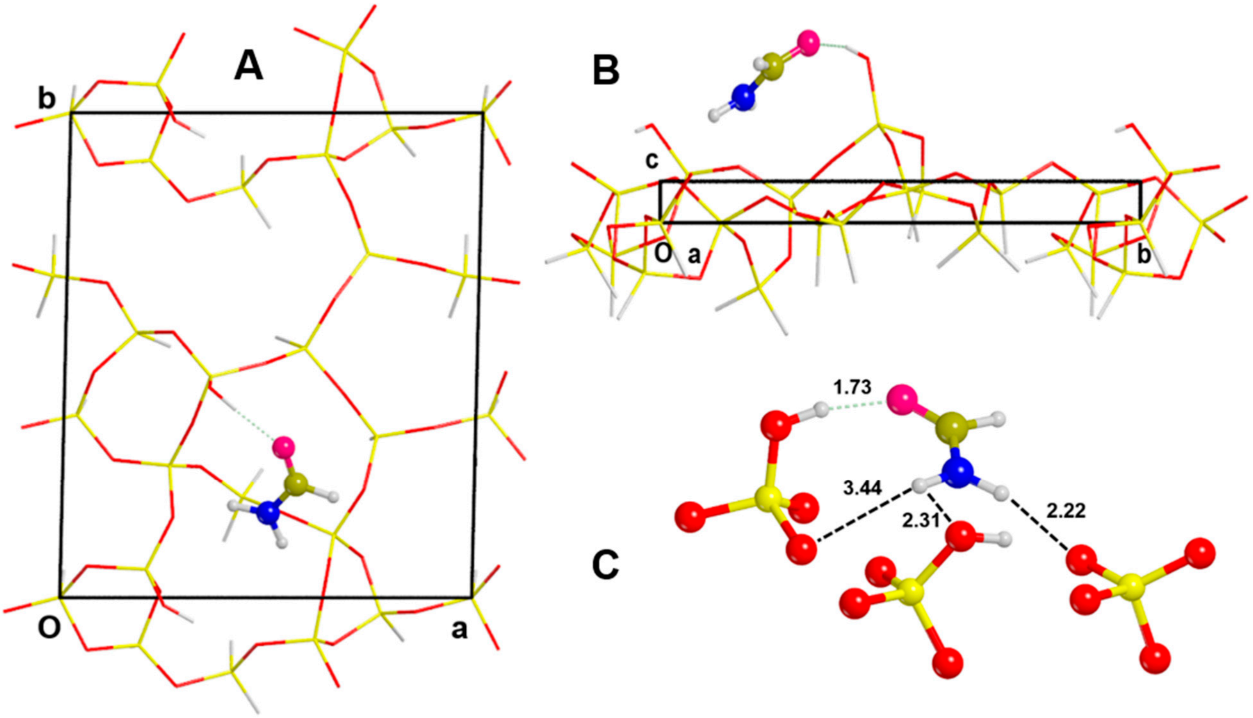

Figure S3. Relaxed geometry of the SiO_2–FA3 model: **(A)** top view; **(B)** side view (along *a* axis); and **(C)** detail of specific interaction and key distances (given in Å). Formamide oxygen is reported in pink, carbon in dark yellow, nitrogen in blue, hydrogen in gray, silicon in bright yellow, SiO_2 oxygen in red.

Table S3. Key structural and spectroscopic features for the SiO_2–FA3 model. Distances are given in Å, frequencies in cm^{-1}. The ν(C=O) and δ(NH_2) frequencies have been scaled with separated scale factors.

**SiO_2–FA3**	
d(C=O)	1.233
ν(C=O)	1711
δ(NH_2)	1600

SiO_2–FA4

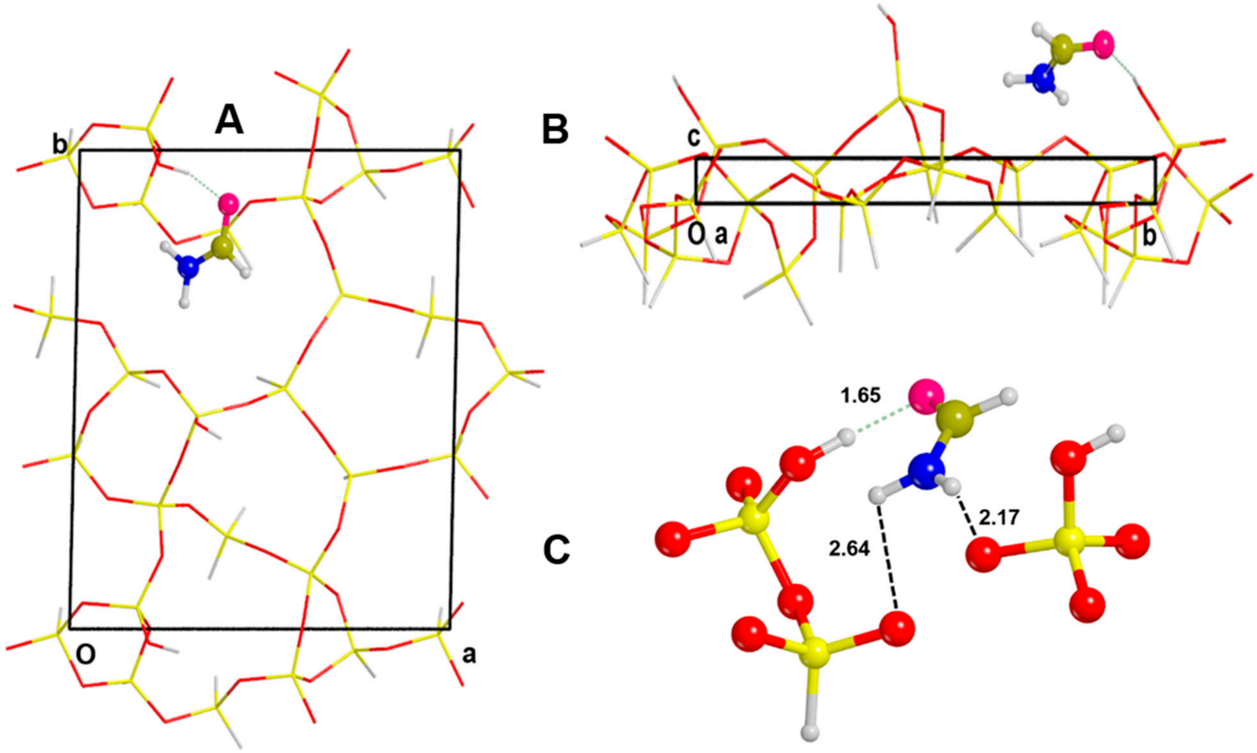

Figure S4. Relaxed geometry of the SiO_2–FA4 model: **(A)** top view; **(B)** side view (along *a* axis); and **(C)** detail of specific interaction and key distances (given in Å). Formamide oxygen is reported in pink, carbon in dark yellow, nitrogen in blue, hydrogen in gray, silicon in bright yellow, SiO_2 oxygen in red.

Table S4. Key structural and spectroscopic features for the SiO_2–FA4 model. Distances are given in Å, frequencies in cm^{-1}. The ν(C=O) and δ(NH_2) frequencies have been scaled with separated scale factors.

	SiO_2–FA4
d(C=O)	1.242
ν(C=O)	1684
δ(NH_2)	1603

SiO_2–FA5

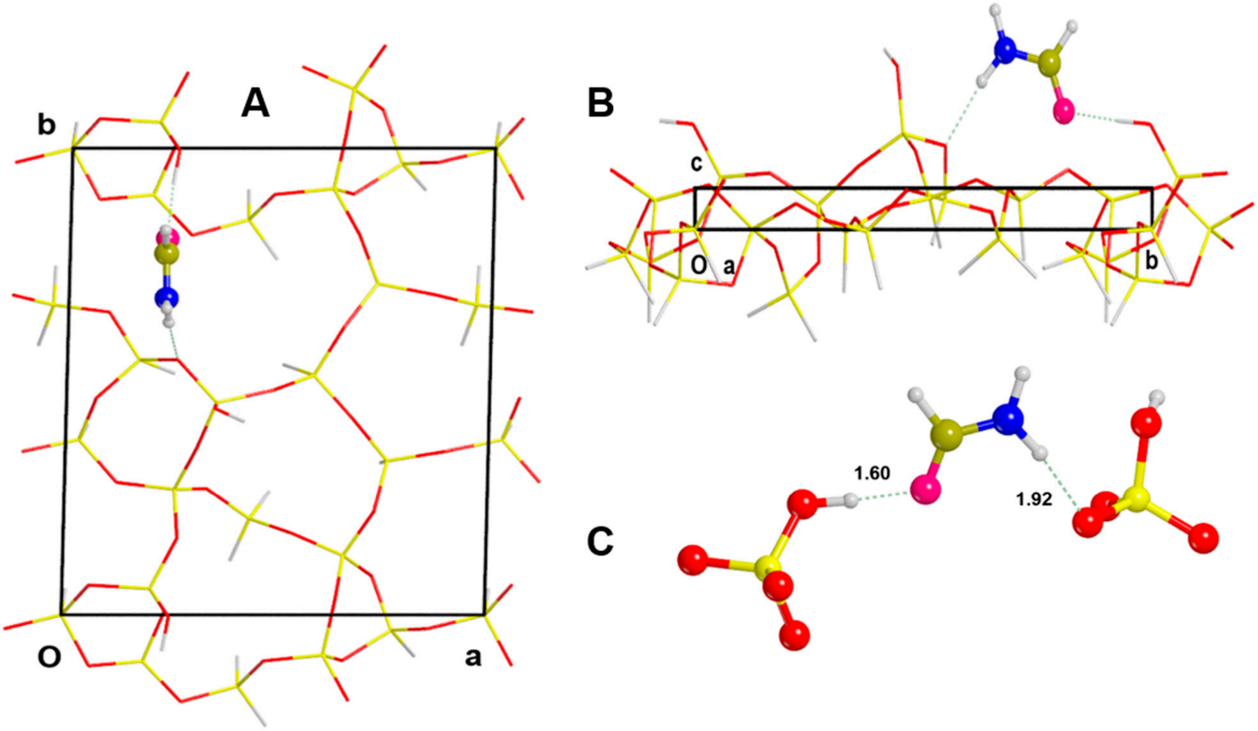

Figure S5. Relaxed geometry of the SiO_2–FA5 model: **(A)** top view; (**B)** side view (along *a* axis); and **(C)** detail of specific interaction and key distances (given in Å). Formamide oxygen is reported in pink, carbon in dark yellow, nitrogen in blue, hydrogen in gray, silicon in bright yellow, SiO_2 oxygen in red.

Table S5. Key structural and spectroscopic features for the SiO_2–FA5 model. Distances are given in Å, frequencies in cm^{-1}. The ν(C=O) and δ(NH_2) frequencies have been scaled with separated scale factors.

SiO_2–FA5	
d(C=O)	1.237
ν(C=O)	1703
δ(NH_2)	1585

SiO_2–2FA1

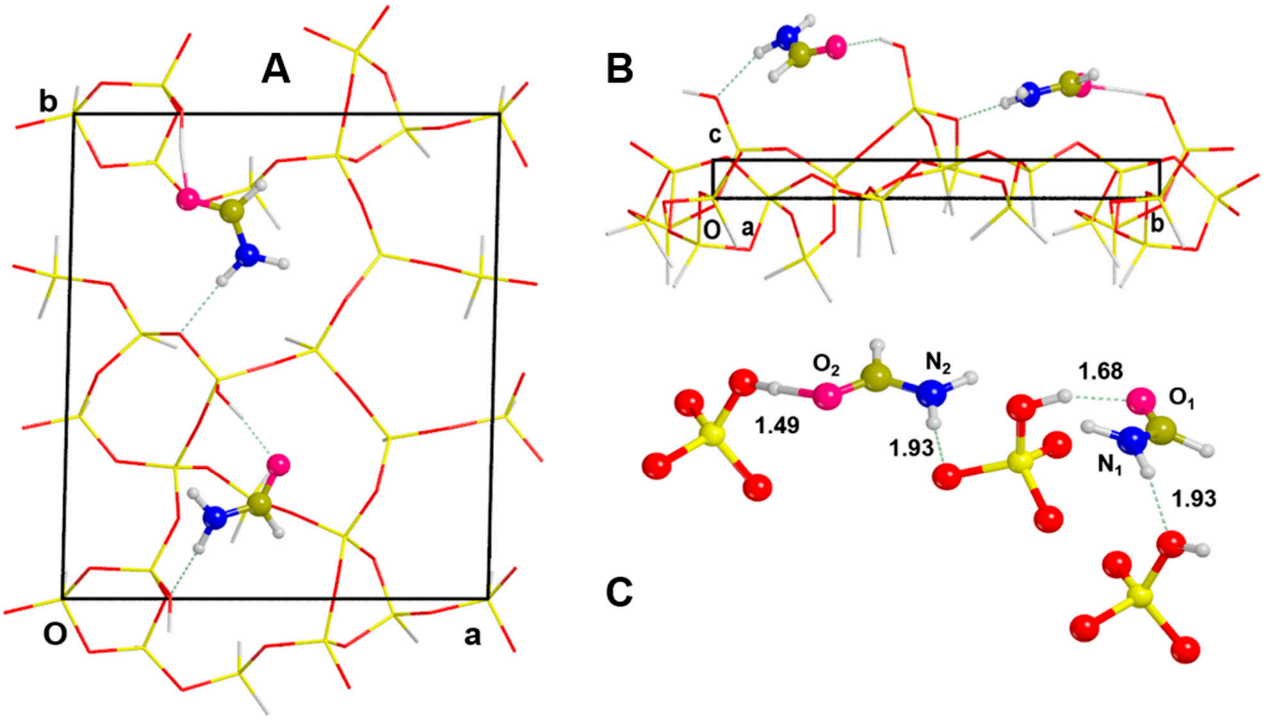

Figure S6. Relaxed geometry of the SiO_2–2FA1 model: **(A)** top view; (**B)** side view (along *a* axis); and **(C)** detail of specific interaction and key distances (given in Å). Formamide oxygen is reported in pink, carbon in dark yellow, nitrogen in blue, hydrogen in gray, silicon in bright yellow, SiO_2 oxygen in red.

Table S6. Key structural and spectroscopic features for the SiO_2–2FA1 model. Distances are given in Å, frequencies in cm^{-1}. The ν(C=O) and δ(NH_2) frequencies have been scaled with separated scale factors.

**SiO_2–2FA1**	
d(C=O)$_1$	1.2418
d(C=O)$_2$	1.2445
ν(C=O)$_1$	1676
ν(C=O)$_2$	1690
δ(NH_2)$_1$	1612
δ(NH_2)$_2$	1593

SiO_2–2FA2

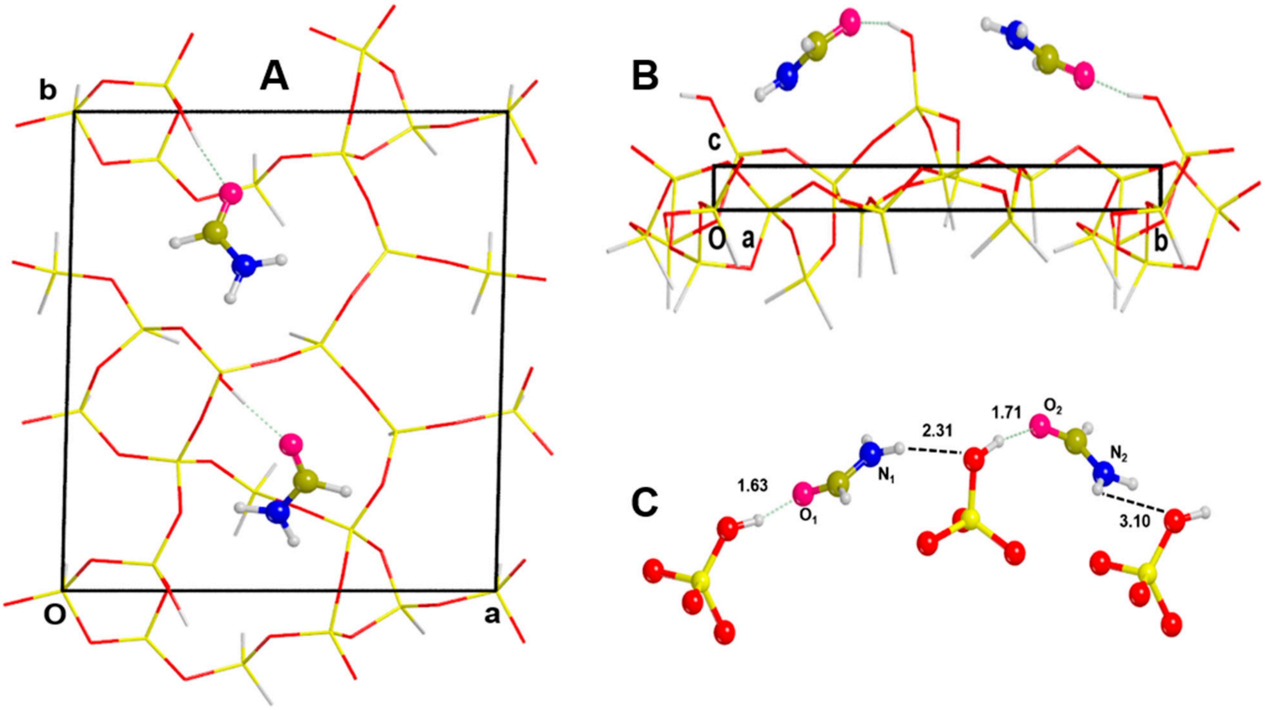

Figure S7. Relaxed geometry of the SiO_2–2FA2 model: **(A)** top view; (**B)** side view (along *a* axis); and **(C)** detail of specific interaction and key distances (given in Å). Formamide oxygen is reported in pink, carbon in dark yellow, nitrogen in blue, hydrogen in gray, silicon in bright yellow, SiO_2 oxygen in red.

Table S7. Key structural and spectroscopic features for the SiO_2–2FA2 model. Distances are given in Å, frequencies in cm^{-1}. The ν(C=O) and δ(NH_2) frequencies have been scaled with separated scale factors.

**SiO_2–2FA2**	
$d(C=O)_1$	1.241
$d(C=O)_2$	1.237
$\nu(C=O)_1$	1686
$\nu(C=O)_2$	1707
$\delta(NH_2)_1$	1592
$\delta(NH_2)_2$	1605

SiO_2–2FA3

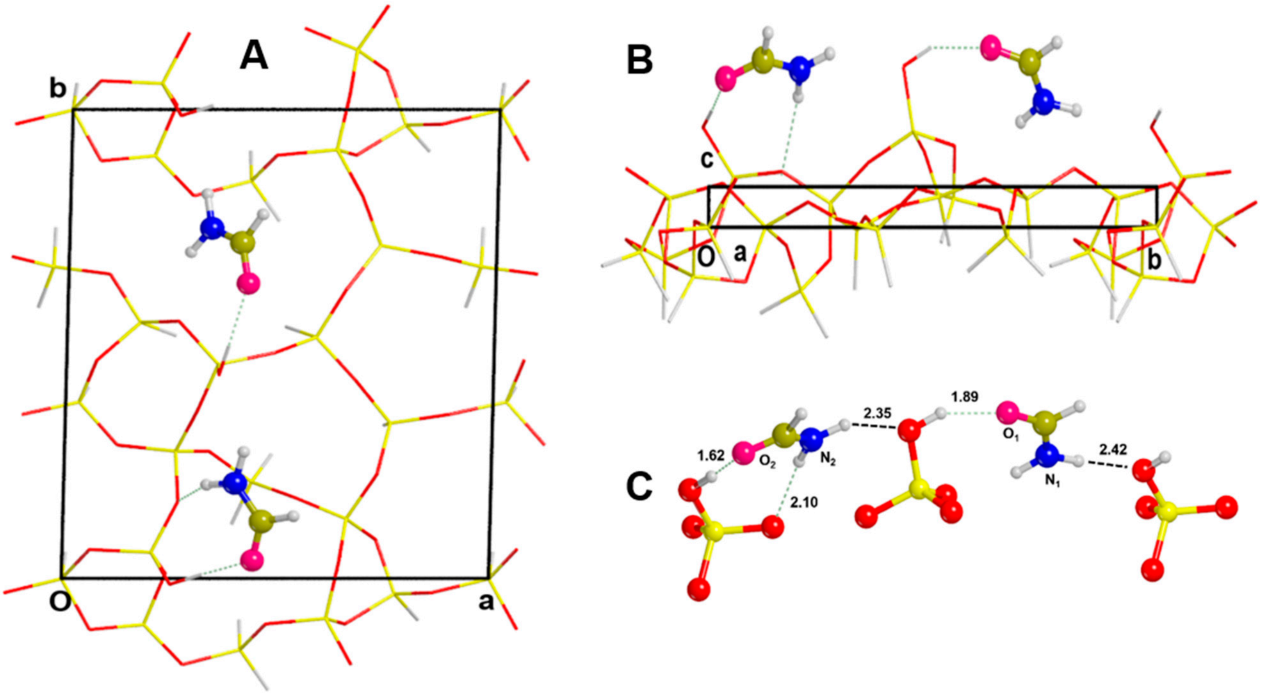

Figure S8. Relaxed geometry of the SiO_2–2FA3 model: **(A)** top view; (**B)** side view (along *a* axis); and **(C)** detail of specific interaction and key distances (given in Å). Formamide oxygen is reported in pink, carbon in dark yellow, nitrogen in blue, hydrogen in gray, silicon in bright yellow, SiO_2 oxygen in red.

Table S8. Key structural and spectroscopic features for the SiO_2–2FA3 model. Distances are given in Å, frequencies in cm^{-1}. The ν(C=O) and δ(NH_2) frequencies have been scaled with separated scale factors.

**SiO_2–2FA3**	
$d(C=O)_1$	1.238
$d(C=O)_2$	1.244
$\nu(C=O)_1$	1673
$\nu(C=O)_2$	1698
$\delta(NH_2)_1$	1582
$\delta(NH_2)_2$	1595

SiO_2–Dim1

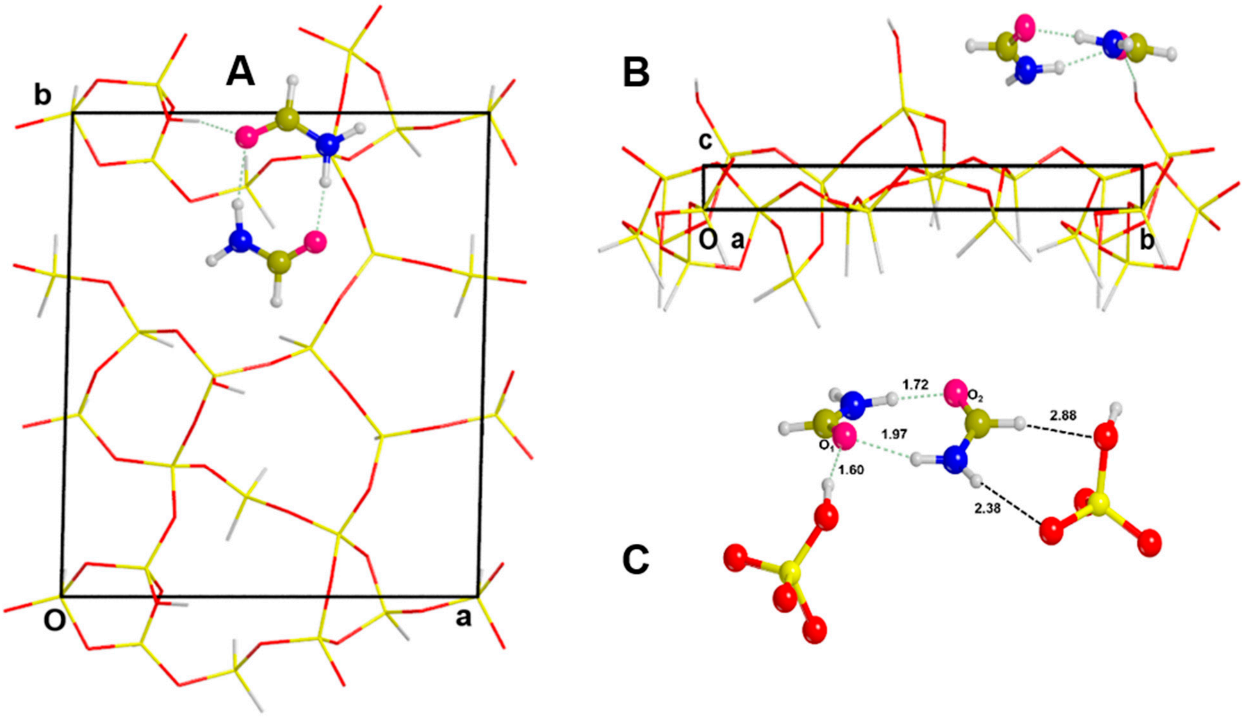

Figure S9. Relaxed geometry of the SiO_2–Dim1 model: **(A)** top view; (**B)** side view (along *a* axis); and **(C)** detail of specific interaction and key distances (given in Å). Formamide oxygen is reported in pink, carbon in dark yellow, nitrogen in blue, hydrogen in gray, silicon in bright yellow, SiO_2 oxygen in red.

Table S9. Key structural and spectroscopic features for the SiO_2–Dim1 model. Distances are given in Å, frequencies in cm^{-1}. The ν(C=O) and δ(NH_2) frequencies have been scaled with separated scale factors.

	**SiO_2–Dim1**
$d(C{=}O)_1$	1.245
$d(C{=}O)_2$	1.240
$\nu(C{=}O)_1$	1703
$\nu(C{=}O)_2$	1683
$\delta(NH_2)_1$	1598
$\delta(NH_2)_2$	1580

SiO_2–Dim2

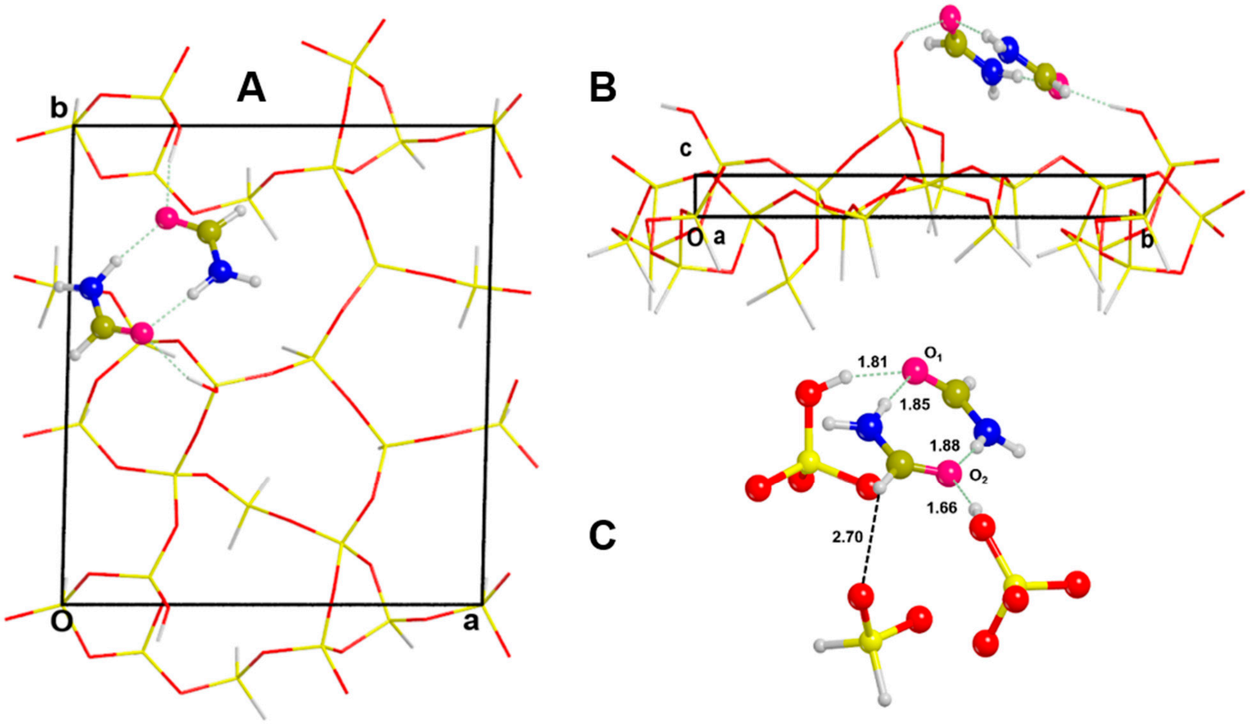

Figure S10. Relaxed geometry of the SiO_2–Dim2 model: **(A)** top view; (**B)** side view (along *a* axis); and **(C)** detail of specific interaction and key distances (given in Å). Formamide oxygen is reported in pink, carbon in dark yellow, nitrogen in blue, hydrogen in gray, silicon in bright yellow, SiO_2 oxygen in red.

Table S10. Key structural and spectroscopic features for the SiO_2–Dim2 model. Distances are given in Å, frequencies in cm^{-1}. The ν(C=O) and δ(NH_2) frequencies have been scaled with separated scale factors.

**SiO_2–Dim2**	
$d(C=O)_1$	1.240
$d(C=O)_2$	1.245
$\nu(C=O)_1$	1697
$\nu(C=O)_2$	1672
$\delta(NH_2)_1$	1612
$\delta(NH_2)_2$	1594

SiO_2–Dim3

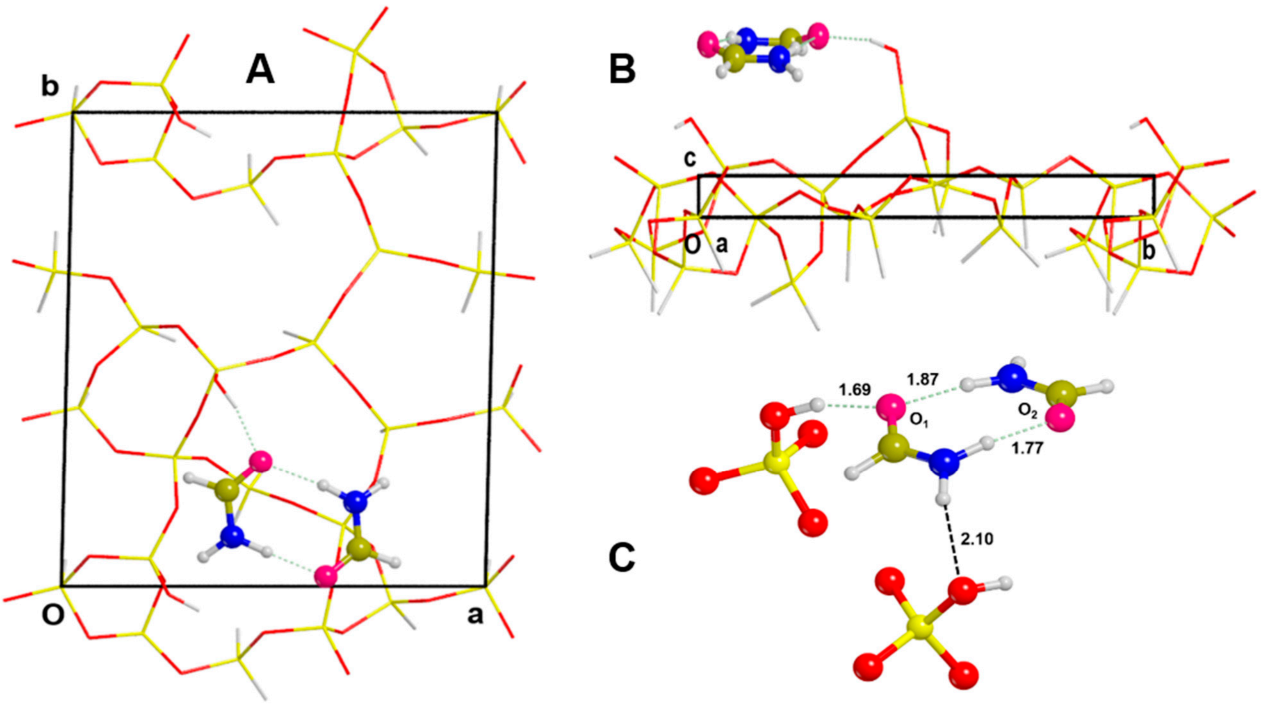

Figure S11. Relaxed geometry of the SiO_2–Dim3 model: **(A)** top view; (**B)** side view (along *a* axis); and **(C)** detail of specific interaction and key distances (given in Å). Formamide oxygen is reported in pink, carbon in dark yellow, nitrogen in blue, hydrogen in gray, silicon in bright yellow, SiO_2 oxygen in red.

Table S11. Key structural and spectroscopic features for the SiO_2–Dim3 model. Distances are given in Å, frequencies in cm^{-1}. The ν(C=O) and δ(NH_2) frequencies have been scaled with separated scale factors.

**SiO_2–Dim3**	
$d(C{=}O)_1$	1.254
$d(C{=}O)_2$	1.240
$\nu(C{=}O)_1$	1708
$\nu(C{=}O)_2$	1678
$\delta(NH_2)_1$	1624
$\delta(NH_2)_2$	1613

SiO_2–Dim4

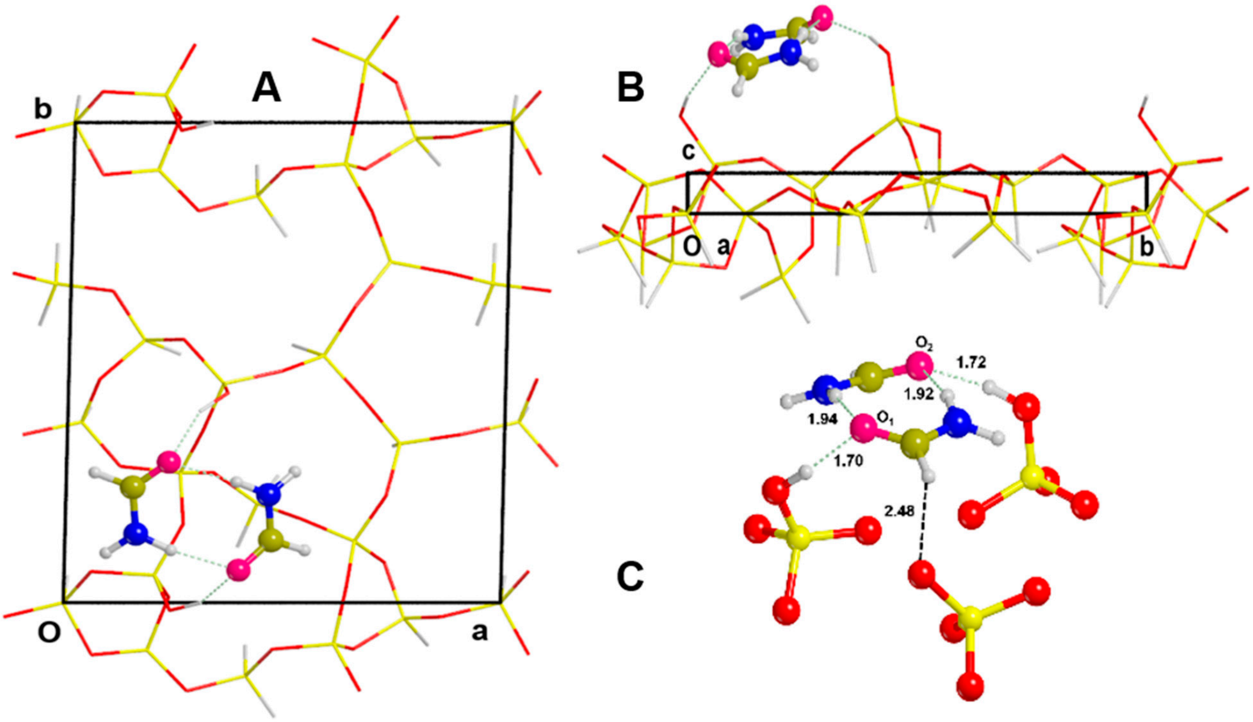

Figure S12. Relaxed geometry of the SiO_2–Dim4 model: **(A)** top view; (**B)** side view (along *a* axis); and **(C)** detail of specific interaction and key distances (given in Å). Formamide oxygen is reported in pink, carbon in dark yellow, nitrogen in blue, hydrogen in gray, silicon in bright yellow, SiO_2 oxygen in red.

Table S12. Key structural and spectroscopic features for the SiO_2–Dim4 model. Distances are given in Å, frequencies in cm^{-1}. The ν(C=O) and $\delta(NH_2)$ frequencies have been scaled with separated scale factors.

	SiO_2–Dim4
$d(C{=}O)_1$	1.248
$d(C{=}O)_2$	1.244
$\nu(C{=}O)_1$	1701
$\nu(C{=}O)_2$	1669
$\delta(NH_2)_1$	1593
$\delta(NH_2)_2$	1586

The energies of adsorption of two formamide molecules to give directly the HC models were computed. The following process has been considered:

SiO_2 + 2 formamide → SiO_2···2 formamide (S1)

The energetic parameter calculated accordingly are reported in Table S13.

Table S13. Adsorption energies (ΔE^c, with explicit dispersive contributions reported in brackets), enthalpies (ΔH^c) and Gibbs free energies (ΔG^c) for the SiO_2-formamide adducts high coverage models generated by double adsorption over the pristine SiO_2 surface. The energy values are calculated according to the reaction reported in eq. 2. All the reported energy values are expressed in kJmol^{-1} have been BSSE corrected through the counterpoise method. The Bolzmann population (p) calculated from the ΔG^c at STP conditions (298.15 K, 1 atm) is reported too.

Model	ΔE^c (ΔE disp)	ΔH^c	ΔG^c	p
SiO_2-2FA1	-150.7 (-55.8)	-136.8	-35.6	0.00
SiO_2-2FA2	-149.4 (-47.8)	-135.9	-40.2	0.01
SiO_2-2FA3	-160.4 (-44.3)	-146.7	-51.8	0.91
SiO_2-Dim1	-139.8 (-51.6)	-126.7	-26.6	0.00
SiO_2-Dim2	-166.5 (-51.5)	-151.4	-45.8	0.08
SiO_2-Dim3	-144.7 (-46.7)	-130.2	-31.5	0.00
SiO_2-Dim4	-151.6 (-48.7)	-136.1	-35.3	0.00
